# Editors’ Book Table

**Published:** 1871-01

**Authors:** 


### Editor's Book Table.

[NOTE.—All works reviewed in the columns of the CHICAGO MEDICAL JOURNAL may be found in the extensive stock of W. B. KEEN & COOKE, whose catalogue of Medical Books will be sent to any address upon request.]

*Galvano-Therapeutics. The Physiological and Therapeutical Action of the Galvanic Current upon the Acoustic, Optic, Sympathetic and Pneumogastric Nerves.* By WILLIAM B. NEFTEL, M.D. New York: D. A. Appleton & Co., 90, 92 and 94 Grand street. 1871. Pp. 191.

Dr. Neftel has had unusual opportunities for observation in the great hospitals of Europe, especially at St. Petersburg, and has devoted the greater part of his attention to the study of nervous diseases. He announces that he is now engaged in the preparation of an elaborate work in two volumes, the first being a treatise on diseases of the nervous system, and the second treating of the galvanic current in its relation to physiology, medicine and surgery. The present essay is a part of the last work, and appears now in print at the request of several aural surgeons and other professional gentlemen.

A perusal of this little book is recommended to those who desire further insight of this obscure subject, or to make practical application of electro-therapeutics.

*Physics and Physiology of Spiritualism.* By WILLIAM A. HAMMOND, M.D., Professor of Diseases of the Mind and Nervous System, and Clinical Medicine, in Bellevue Hospital Medical College, etc., etc. New York: D. Appleton & Company, 1871. Pp. 86.

The basis of this monograph is an essay contributed to the North American Review in April, 1870. It has been thoroughly revised, with large additions.

The author, as is well known, belongs to the advanced school of physiologists who are not "fettered by theological and metaphysical dogmas." He recognizes mind as a force the result of nervous action. Of its several qualities, consciousness resides exclusively in the brain, but the others are developed with more or less intensity by other parts of the nervous system; and in the fact that the spinal cord and sympathetic ganglia are possessed of mental power he finds an explanation of some of the most striking phenomena of spiritualism.

Dr. H. refers to animal electricity and magnetism—gives an amusing illustration of one Dr. Von Vleck's reproduction, by sleight-of-hand, of the performances of the Davenport Brothers, and then discusses more at length natural and artificial somnambulism. In hypnotism the phenomena are not those of pure somnambulism, but include, in greater or less degree, hysteria, catalepsy and ecstacy. These conditions are then discussed and illustrated. The dangerous voluntary power possessed by some persons, of voluntarily producing hallucination of various kinds, is cited.

The alleged spiritual phenomenon of levitation, including the rising in the air of tables, chairs, musical instruments and even of the human body itself, is then examined, and both historically in witchcraft, and at the present time in spiritualism, are referred to either (or all) hallucination, legerdemain, or actual fraud.

Admitting all the assumed phenomena of "spiritualism," the author rationally concludes that "there would be no proof that spirits had anything to do with them." On the contrary, the hypothesis of spirits is the least plausible which could be suggested. The phenomena and the explanation have nothing in common.

For ourselves, we have to say of books of this sort, that while they may help to keep people from falling into the quagmires of spiritualism, they will not extricate any of its unfortunate victims —for, "men are never reasoned out of opinions they were never reasoned into."

*Specific Medication and Specific Medicines.* By JOHN M. SCUDDER, M. D., Professor of the Principles and Practice of Medicine in the Eclectic Medical Institute (Cincinnati), etc., etc. Cincinnati: Wilstach, Baldwin & Co., Printers. 1870. Pp. 253.

The larger portion of this treatise has appeared in monthly articles in the *Eclectic Medical Journal*, and hence will require no introduction to the readers of that paper. The author flatly contradicts the dogma, "There are no specifics in medicine," and yet claims that his theory has "no alliance with the homœopathic specific medication." *Ait:* "I contend that a drug is a specific remedy: first, because it influences uniformly and directly the part or function diseased; and second, because it *opposes* such diseased action. I would, therefore, write the law of cure *opponens opponenda*, instead of *similia similibus*.

The author laudably lays it down as "an axiom from which it is never safe to depart, that—No medicine should be given, unless the pathological condition and the indications for its use are clearly defined. It is much better to employ a *placebo*, than run the risk of doing harm by medication." In which we pretty much agree with him.

The action and uses of four or five hundred (mainly vegetable) articles are described, as illustrative of the author's views, and the potency of specifics, particularly those which rejoice the hearts of our Eclectic (late "Botanical") fellow-citizens, are concisely written down. We confess to a latent fear that the relations of some, at least, of the number to "the pathological condition" are not "clearly defined."

*The Transactions of the American Medical Association.* Instituted 1847. Vol. XXI. Philadelphia: Printed for the Association. Collins, Printers, 705 Jayne street. 1870. Pp. 612.

The proceedings of the Association having been quite fully detailed in the No. of the JOURNAL for June last, need no further notice at this time. The current volume is produced in good style, unimpeachable typography, excellent paper, and the essays on Median Lithotomy, Plastic Surgery, Blood Corpuscle, and Aneurism, (the latter, especially) well illustrated.

The report of R. C. Hamill, M.D., on the Diseases of Illinois, has prefixed an outline map of Chicago, to render clear his exposition of the prevailing diseases of this city.

The next meeting of the Association, it will be recollected, is appointed at San Francisco.

*A Practical and Systematic Treatise on Fractures and Dislocations.* By A. JACKSON HOWE, M.D., Professor of Anatomy in the Eclectic Medical Institute. Cincinnati: Chas. F. Wilstach & Co. 1870. 8vo, pp. 424.

With good type, tolerably fair imprint, poor paper, and exceedingly crude wood cuts, the book is not particularly creditable to Cincinnati or the publishers. But its artistic merits are in harmony with the author's efforts, who, while he has produced another volume of so-called "Eclectic" medical literature, and thus fulfilled undoubtedly his purpose, has, in the field of science, achieved the same degree of success that his publishers have in their department of labor and enterprise.

